# Targeted suppression of *HO-2* gene expression impairs the innate anti-inflammatory and repair responses of the cornea to injury

**Published:** 2011-04-29

**Authors:** Lars Bellner, Kiran A. Patil, Kirkland Castellano, Adna Halilovic, Michael W. Dunn, Michal Laniado Schwartzman

**Affiliations:** 1 Department of Pharmacology, New York Medical College, Valhalla, NY; 2 Department of Ophthalmology, New York Medical College, Valhalla, NY

## Abstract

**Purpose:**

Heme oxygenase (*HO*)-2 is highly expressed in the corneal epithelium and is a component of the heme oxygenase system that represents an intrinsic cytoprotective and anti-inflammatory system based on its ability to modulate leukocyte migration and to inhibit expression of inflammatory cytokines and proteins via its products biliverdin/bilirubin and carbon monoxide (CO). We have shown that in *HO-2* null mice epithelial injury leads to unresolved corneal inflammation and chronic inflammatory complications including ulceration, perforation and neovascularization. In this study, we explore whether a localized corneal suppression of *HO-2* is sufficient for disrupting the innate anti-inflammatory and repair capability of the cornea.

**Methods:**

Silencing hairpin RNA (shRNA) against *HO-2* was administered subconjunctivally (100 ng/eye) as well as topically (100 ng/eye) starting one day before corneal epithelial debridement and once daily, thereafter. The corneal epithelium was removed using an Alger Brush in anesthetized mice. Re-epithelialization was assessed by fluorescein staining using a dissecting microscope and image analysis. Inflammatory response was quantified by myeloperoxidase activity. Levels of mRNA were measured by RT–PCR.

**Results:**

Local injection of *HO-2*-specific shRNA led to a 50% reduction in corneal *HO-2* mRNA. Administration of *HO-2*-specific shRNA delayed corneal re-epithelialization when compared with the control shRNA-treated group by 14%, 20%, and 12% at days 3, 4, and 7 after injury, respectively (n=18–24). The observed delay in the wound repair process in *HO-2* shRNA treated mice was accompanied by a threefold and 3.5 fold increase in the neovascular response at days 4 and 7 after injury. Further, local knockdown of *HO-2* lead to an aberrant chronic inflammatory response, as shown by presence of high numbers of inflammatory cells still present in the cornea at day 7 after injury; 1.04±0.45×10^6^ in *HO-2* knockdown mice versus 0.14±0.03×10^6^ inflammatory cells in control mice. Matrix metalloproteinase-2 (*MMP-2*) but not *MMP-9* increased following injury and remained elevated in the injured corneas of the *HO-2* shRNA-treated eyes.

**Conclusions:**

Corneal knockdown of *HO-2* via local administration of *HO-2*-specific shRNA leads to delayed re-epithelialization, increased neovascularization and an aberrant inflammatory response similar to what is observed in the *HO-2* null mouse. The elevated *MMP-2* expression may contribute to the increase in neovascularization in corneas in which *HO-2* expression is suppressed.

## Introduction

The epithelium is the outermost layer of the cornea and primarily functions as a protective barrier to avoid fluid loss and invasion of the eye by pathogens and, through its interaction with the tear film, forms an absolutely smooth and transparent refractive surface. The response of the corneal epithelium to insults is rapid and consists of several consecutive steps starting with immediate migration of the remaining epithelial cells to cover the wound area followed with proliferation and upward movement of cells to form a multilayered functional structure, all processes driven by growth factors and other factors released into the injured area by epithelial cells, keratinocytes and to some extent by inflammatory cells invading the injured cornea [1,2].

The heme oxygenase (HO) system has emerged as a fundamental endogenous cytoprotective (anti-oxidative) and anti-inflammatory system in many tissues. HO catalyzes the degradation of free heme to biliverdin and carbon monoxide (CO), a reaction that is equally performed by the inducible as well as the constitutive HO isoforms, HO-1 and HO-2, respectively [3]. The mechanisms by which HO affords cytoprotection are thought to be attributed to the elimination of excess cellular heme as well as the enzymatic products of the HO system, i.e., CO and bilirubin. The characteristics of HO-1 as an inducible enzyme support the view that it is the primary component of the cytoprotective action exerted by the HO system. Indeed, upregulation of HO-1 suppresses the inflammatory response by either attenuating the expression of adhesion molecules and, thus, inhibiting leukocyte recruitment [4,5], by repressing the induction of cytokines and chemokines [6-10], or by inhibiting pro-inflammatory hemoproteins such as cyclooxygenase (COX)-2 and cytochrome P450 4B1 (CYP4B1) [11-14]. On the other hand, HO-1 deficiency is associated with a chronically inflamed state and increased leukocyte recruitment as reported in both humans [15,16] and in mice [17,18] null for the *HO-1* gene.

*HO-2*, by virtue of its constitutive and relatively constant expression in most tissues, has received less attention. However, its distinct properties including constitutive expression, activation by phosphorylation [19,20] and additional binding site for heme [21] set it apart from HO-1. Furthermore, the demonstration that HO-2 is cytoprotective without degrading heme [22,23] implies that HO-2 participates not only in maintaining heme homeostasis but also in cellular defense mechanisms against injury. To this end, studies using *HO-2* null mice have demonstrated an increased susceptibility to hyperoxic injury in the lung [24] and to oxidative and ischemic injury in the brain [25,26].

In a series of studies, we have shown that the cornea of human, rabbit and mouse exhibits HO activity and expresses *HO-1* in response to injury and oxidative stress in vitro and in vivo [27-30] and that further induction of *HO-1* alleviates injury-induced ocular surface inflammation and accelerates corneal wound healing [27]. Recently, we showed that *HO-2* displays a prominent constitutive expression in the cornea that is localized primarily to the corneal epithelium [29] and is the main contributor of HO activity in the healthy cornea. The function of HO-2 in the avascular cornea is largely unknown. However, recent studies using *HO-2* null mice implicates it as a key component of the corneal inflammatory and repair response. In these studies, we showed that deletion of the *HO-2* gene markedly impairs the inflammatory and reparative response of the cornea to epithelial injury [28,31] and in a model of suture induced neovascularization [29]. Hence, HO-2 deficiency leads to unresolved corneal inflammation and chronic inflammatory complications including ulceration, perforation and neovascularization. Importantly, the outcomes of this deficiency was shown to be reversed in part by supplementation of the HO metabolic product, biliverdin [31].

Based on these studies and the finding of a substantial expression of *HO-2* in the normal corneal epithelium [28,29], it is reasonable to assume a functional role for the epithelial *HO-2* in the regulation of corneal homeostasis. However, the use of *HO-2* null mice does not exclude the possibility of systemic influence of *HO-2* deletion on the response of the cornea to injury. In this study, we used plasmid DNA encoding *HO-2* specific and non-specific shRNAs to examine whether local knockdown of the *HO-2* gene interferes with corneal wound healing in vivo.

## Methods

### Animal experimentation

All animal experiments were performed following an institutionally approved protocol in accordance with the National Institutes of Health Guide for the Care and Use of Laboratory Animals. Mice (C57bl6; Jackson Laboratories, Bar Harbor, ME) were anesthetized with ketamine (50 mg/kg) and xylazine (20 mg/kg) intramuscularly and a drop of tetracaine-HCl 0.5% was applied to the eye to deliver local corneal anesthesia before subjecting animals to injury. The corneal epithelium up to the corneal/limbal border was removed using an Algerbrush II with a 0.5-mm corneal rust ring remover (Alber Equipment Co., Lago Vista, TX) as previously described [28]. Plasmids carrying *HO-2* shRNA (200 ng /eye) were mixed with collagen (Atelocollagen, Koken Co. Ltd, Tokyo, Japan) and then administered subconjunctivally adjacent to the limbal border using a Hamilton syringe, (a total of 4 µl shRNA/collagen-mix, 1 µl in each quadrant of the limbal border, was injected) as well as topically (100 ng plasmid/eye in 4 µl) starting one day before corneal epithelial debridement and every other day thereafter. Wound closure and neovascularization were measured at day 2, 4, and 7 after injury. Digital images of the anterior surface were taken with a Zeiss dissecting microscope using Axiovision 4.5 software and analyzed by Axiovision 4.5 software (Carl Zeiss, Göttingen, Germany). Mice were euthanized at the indicated time points, eyes were removed, and corneas, free of conjunctival tissue, were dissected and processed for selected analyses.

### shRNA plasmids

The shRNAs were obtained from OriGene Technologies, Inc. (Rockville, MD). The shRNA expression cassette consists of a 29 bp target gene specific sequence, a 7 bp loop, and another 29 reverse complementary sequence, all under a human U6 promoter. A termination sequence (TTTTTT) is located immediately downstream of the second 29 bp reverse complementary sequence to terminate the transcription by RNA Pol III. The 29 bp gene-specific sequence was sequence-verified to ensure its match to the target gene. The *HO-2* oligonucleotide sequence was 5′-TGA GTC AGA GAA GAA CTC TAT GGC ACC AG’3′. A plasmid containing shRNA directed against green fluorescent protein (GFP) was used as a negative control. The shRNA plasmids were transformed and amplified in *E. coli* according to the manufacturer’s instructions. Plasmid DNA was extracted using the Plasmid Midi Prep (Qiagen, Valencia, CA).

### Histology

Dissected corneas were washed twice with PBS and fixed in 4% paraformaldehyde-PBS for 1 h at 4 °C. Corneas were washed five times with PBS, placed in 30% sucrose for 24 h and embedded in optimal cutting temperature (OCT) compound (Sakura Finetek, Torrence, CA). Cryostat sections were cut transversely into 5–7 µm thick sections, stained with Hematoxylin-Eosin and mounted on microscopic slides in Cytoseal XYL (Richard-Allan Scientific, Kalamazoo, MI).

### Myeloperoxidase (MPO) activity

Measurement of MPO activity was used to quantify polymorphonuclear cells (PMNs) in dissected corneas as previously described [28]. In brief, tissues were homogenized in potassium phosphate buffer (pH 6.0) containing 0.5% hexadecyltrimethylammonium bromide, followed by three cycles of sonication and freeze–thaw. The particulate matter was removed by centrifugation, and MPO activity in the supernatant was measured by spectrophotometry using o-dianisidine dihydrochloride reduction as a colorimetric indicator. Calibration curves for conversion of MPO activities to PMN number were established with PMNs that were collected from zymosan A-induced peritonitis in mice.

### Real-time polymerase chain reaction (PCR)

Corneas were aseptically dissected from eyes and cleaned in sterile PBS (4 °C) under a dissecting microscope to remove all non-corneal tissue. Total RNA was isolated using the RNeasy^®^ Plus Micro Kit (Qiagen, Germantown, MD) and RNA was quantitated using a Nanodrop spectrophotometer (Nanodrop Technologies, Wilmington, DE). Reverse transcription reaction of total RNA was performed using the qScript™ cDNA Synthesis Kit (Quanta BioSciences, Inc., Gaithersburg, MD) according to the manufacturer’s instruction. Quantitative real-time PCR was performed using PerfeCTa™ SYBR^®^ Green FastMix™ Kit (Quanta BioSciences, Inc.) and the Mx3000 real-time PCR system (Stratagene, La Jolla, CA). Specific primers, purchased from Gene Link™ (Hawthorne, NY), were designed using Primerbank based on published sequences (GenBank) and were as follows: 18S sense, 5′-TGT CTC AAA GAT TAA GCC ATG CAT-3′ and anti-sense, 5′-AAC CAT AAC TGA TTT AAT GAG CCA TTC-3′; *HO-1* sense, 5′-TCC AGA CAC CGC TCC TCC AG-3′ and anti-sense, 5′-GGA TTT GGG GCT GCT GGT TTC-3′; *HO-2* sense, 5′-TAC TTC ACA TAC TCA GCC CT-3′ and anti-sense, 5′-ATG GGC CAC CAG CAG CTC TG-3′; *MMP-2* sense, 5′-GAC CTT GAC CAG AAC AAC ATC-3′ and antisense, 5′-CAT CCA CGG TTT CAG GGT CC-3′; *MMP-9* sense, 5′-TGC CCA TTT CGA CGA CGA C-3′ and antisense, 5′-GTG CAG GCC GAA TAG GAG C-3′. Quantitative analysis was performed as previously described [27.29].

### Statistical analysis

Student’s *t*-test was used to evaluate the significance of differences between groups and multiple comparisons were performed by regression analysis and one-way ANOVA. P values less than 0.05 were considered significant. All data are presented as mean±SEM.

## Results

### Local administration of *HO-2* shRNA suppresses corneal *HO-2* but not *HO-1* mRNA expression

**Figure 1:**
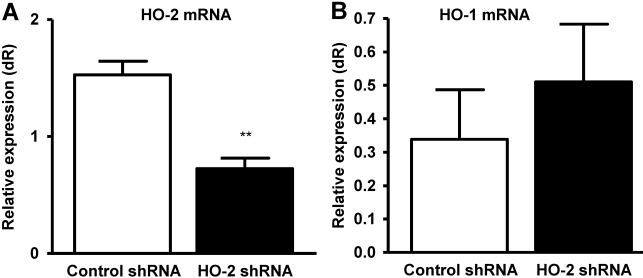
Effect of local *HO-2* knockdown on *HO-2* and *HO-1* mRNA levels. Corneal **A**: *HO-2*, and **B**: *HO-1* mRNA levels after control- and *HO-2*-shRNA-treatment. (**p<0.01 from control-shRNA-treated mice, n=2–4).

*HO-2* is constitutively expressed in the cornea at relatively high levels and its level after injury remains largely unchanged [30]. As seen in Figure 1A, local injection of *HO-2* shRNA into the conjunctiva adjacent to the limbal border caused a 50% decrease in the level of *HO-2* mRNA as compared to injection with control shRNA. The level of *HO-1* following *HO-2* shRNA treatment was largely unaltered (Figure 1B).

### Local suppression of *HO-2* delays corneal wound healing

**Figure 2:**
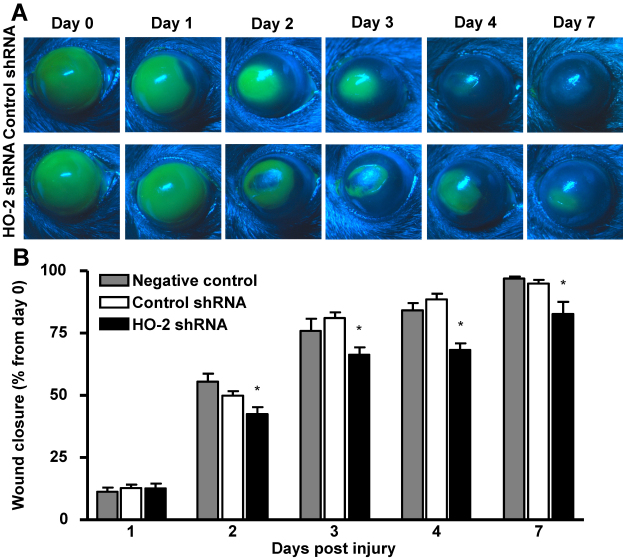
Effect of local *HO-2* knockdown on corneal wound healing. **A**: Fluorescein-stained corneas after injury in control- and *HO-2*-shRNA-treated mice. **B**: Wound closure as percent from day 0 (*p<0.05 from non-treated, and control shRNA treated mice, n=3–24).

Epithelial injury produced a consistent wound (7.03±0.032 mm^2^, n=47) that exhibited a linear rate of re-epithelialization in non-specific (control) shRNA-treated mice with 12.87%±1.31%, 49.87%±1.86%, 81.09%±2.25%, 88.65%±2.20%, and 94.98%±1.45% wound closure at days 1, 2, 3, 4, and 7 after injury, respectively (Figure 2A). the rate of re-epithelialization was not significantly different from untreated mice. In contrast, re-epithelialization of corneal wounds (6.04±0.16 mm^2^, n=4) in *HO-2* shRNA-treated mice was blunted by 0.25%, 7%, 14% 20%, and 12% at days 1, 2, 3, 4, and 7 after injury as compared with mice treated with control shRNA (Figure 2B).

### Local suppression of *HO-2* increases injury-induced corneal neovascularization

**Figure 3:**
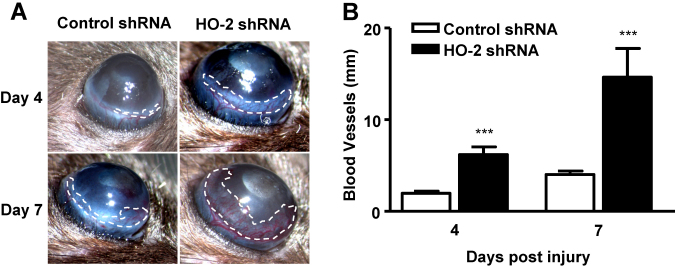
Effect of local *HO-2* knockdown on the injury induced corneal neovascularization. **A**: Photographs showing neovascularization in control- and *HO-2* shRNA-treated mice at days 4, and 7 after epithelial debridement. **B**: Neovascularization expressed as total length of penetrating vessels (***p<0.001 from control-shRNA-treated mice, n=10–21).

We have previously shown that in the *HO-2* null mice epithelial injury causes massive corneal neovascularization [28]. We examined whether localized suppression of corneal *HO-2* expression also increases the degree of neovascularization. As seen in Figure 3A, eyes treated with *HO-2*-specific shRNA showed increased corneal neovascularization; the total vessel length was 6.20±0.83 and 14.63±3.14 mm at days 4 and 7, respectively, compared to 1.97±0.25 and 4.02±0.39 mm at days 4 and 7 in control shRNA-treated mice (n=10–21; Figure 3B).

### *HO-2* suppression alters the inflammatory response in the injured cornea

**Figure 4:**
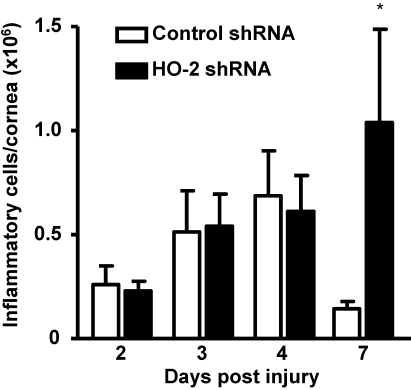
Effect of local *HO-2* knockdown on the injury induced inflammatory response. Corneal MPO activity in control- and *HO-2*-shRNA-treated mice at days 2, 3, 4, and 7 after epithelial debridement (*p<0.05 from control-shRNA-treated mice, n=6–12).

In WT mice, the corneal inflammatory response in response to epithelial injury is characterized by a transient influx of inflammatory cells into the stroma, whereas in the *HO-2* null mice the inflammatory response remains unresolved [28]. In this study, local *HO-2* silencing showed a similar result (Figure 4), causing the number of inflammatory cells to remain elevated at the end of the experimental period of 7 days; the number of neutrophils per cornea, as measured by MPO-activity, being (in millions/cornea) 0.23±0.05, 0.54±0.15, 0.61±0.17, and 1.04±0.45 at days 2, 3, 4, and 7 after injury, correspondingly as compared to 0.26±0.09, 0.51±0.20, 0.69±0.22, and 0.14±0.03 at days 2, 3, 4, and 7 in control shRNA treated mice (n=6–11).

**Figure 5:**
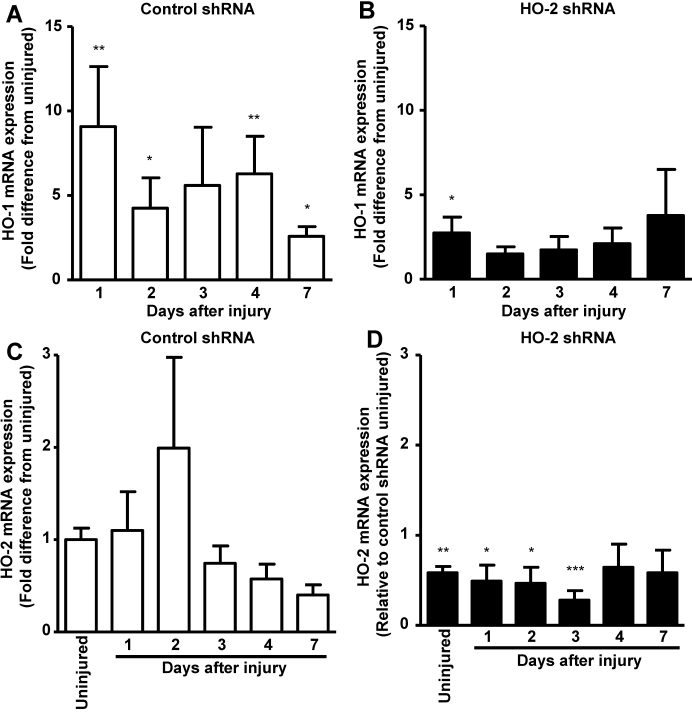
Effect of local *HO-2* knockdown on injury induced *HO-1* mRNA expression and on *HO-2* mRNA expression. Corneal *HO-1* mRNA levels in **A**: control shRNA and **B**: *HO-2*-shRNA-treated corneas 1, 2, 3, 4, and 7 days post epithelial debridement (*p<0.05; **p<0.01 from corresponding uninjured corneas, n=5–7). Corneal *HO-2* mRNA levels in **C**: control shRNA and **D**: *HO-2*-shRHA-treated corneas in uninjured corneas, and 1, 2, 3, 4, and 7 days post epithelial debridement (*p<0.05; **p<0.01; ***p<0.001 from control shRNA-treated uninjured corneas, n=5–10).

Induction of *HO-1* is a rapid and transient cytoprotective response to injury in many tissues including the cornea [28,29,32,33]. We have shown that in the absence of HO-2 this response is compromised in the injured cornea [28,29]. This effect was also seen with local knockdown of *HO-2*. As seen in Figure 5A, in the control shRNA-treated eyes, corneal *HO-1* mRNA levels increased by 10-fold within 24 h after injury, remained elevated by 5–6 fold through day 4 and decreased to levels of 2.5 fold higher than in uninjured corneas. In contrast, in the *HO-2* shRNA-treated eyes, *HO-1* induction in response to injury was markedly blunted. *HO-1* mRNA rose to levels of 2.5 fold higher than uninjured corneas and remained roughly unchanged at levels not significant from those in the uninjured cornea (Figure 5B). The levels of *HO-2* mRNA in the control shRNA- and the *HO-2* shRNA-treated corneas remained largely unchanged throughout the duration of the experiment (Figure 5C,D). The *HO-2* mRNA levels in the *HO-2* shRNA-treated corneas were however significantly reduced as compared to uninjured control shRNA-treated corneas at days 1, 2, and 3, but not at days 4 and 7, possibly due to *HO-2* mRNA brought into the tissue by infiltrating inflammatory cells (Figure 5D).

**Figure 6:**
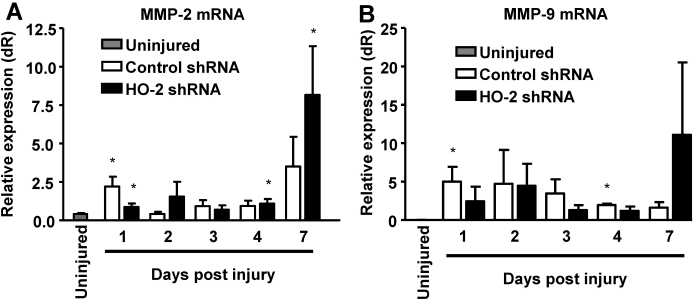
Effect of local *HO-2* knockdown on injury induced *MMP-2* and *MMP-9* mRNA expression. Corneal mRNA levels of **A**: *MMP-2* and **B**: *MMP-9* in uninjured corneas and in control- and *HO-2*-shRNA-treated corneas 2, 4, and 7 days post epithelial debridement (*p<0.05 from uninjured, n=2–5).

Matrix metalloproteinases (MMPs) have been shown to play an important role in epithelial repair and stromal remodeling following injury [34,35]. In a recently published study, we showed that both *MMP-2* and *MMP-9* mRNA are expressed in corneas of *HO-2* null mice, and that the mRNA levels of *MMP-2*, which peaked at day 7 after injury, were significantly lowered in corneas of biliverdin-treated mice, that in addition developed less severe corneal epithelial defects and reduced corneal neovascularization [31]. Therefore, in this study, we examined whether local knockdown of *HO-2* affect the expression of these *MMP*s in response to injury and during wound healing. As seen in Figure 6A,B, corneal *MMP-2* and *MMP-9* mRNA levels were elevated in response to injury with significantly higher levels of *MMP-2* at days 1, 4, and 7 after injury and *MMP-9* at days 1, 3, 4, and 7 after injury, as compared to uninjured corneas. Interestingly, the levels of *MMP-2* and *MMP-9* mRNA seemed higher at day 7 in the corneas of mice treated with *HO-2* shRNA as compared to control shRNA-treated mice (Figure 6A,B).

## Discussion

Inflammation is a vital physiologic response to injury in living tissues and, when tightly controlled, contributes to normal healing and repair; it enables and crucially drives tissue repair by stimulation of resident cells to migrate and proliferate into the wound site, but severely disturbs wound healing processes when it is prolonged or overly exaggerated. Hence, an ordered execution and resolution of inflammation is an essential step toward wound healing; it requires balanced and defined formation of pro- as well as anti-inflammatory signals which orchestrate a complex and well controlled biologic and biochemical process involving tissue cells and cells of the immune system. We have previously identified the *HO-2* as a key anti-inflammatory and cytoprotective signal in the cornea and showed that its deletion impairs the corneal inflammatory and repair response; *HO-2*-deficient corneas respond to injury with exaggerated inflammation and lack of resolution leading to impaired wound healing, perforation, ulceration and neovascularization [28].

The present study was undertaken to evaluate the consequences of local rather than global suppression of *HO-2* to the inflammatory and repair response of the cornea to injury. The intracellular delivery of small interfering/inhibitory RNA is a therapeutic strategy to transiently block gene expression with great specificity and potency and an excellent alternative to other genetic knockdown methods for the analysis of loss-of-function phenotypes; it can be employed locally and at any stage of the experiment. Two silencing RNA strategies utilize either synthetic double stranded RNA or plasmid DNA encoding a shRNA [36,37]; both have been shown to be of great value in knocking down genes in a variety of biologic systems. Both strategies have been used successfully to locally suppress the corneal expression of several genes including vascular endothelial growth factor (*VEGF*) and cytochrome P450 4B1 (*CYP4B1*) in vivo under various experimental conditions [38-43]. We have recently used this strategy to knockdown *HO-2* in human corneal epithelial cells and showed that deficiency in *HO-2* impairs wound closure in vitro [30]. In this study, we used plasmid DNA encoding *HO-2* specific and non-specific shRNAs to examine whether local knockdown of *HO-2* gene interferes with corneal wound healing in vivo and corneal epithelial cell proliferation and migration in vitro. Our results clearly indicate that this approach is working; subconjunctival injection of *HO-2*-specific but not the non-specific control shRNA significantly suppressed *HO-2* mRNA levels in the cornea.

Similar to data obtained with the *HO-2* null mice [28], in situ suppression of *HO-2* in WT mice was associated with attenuation of corneal wound healing. Thus, wound closure was completed by day 7 after injury in corneas treated with nonspecific shRNA, whereas, wound healing was significantly impaired in *HO-2* specific shRNA-treated corneas showing a closure of the wound of only about 70% at day 7 after injury. The impaired healing was also associated with increased corneal neovascularization, a finding consistent with previous studies linking deficiency in *HO-2* expression to increased angiogenesis [28,29,31]. In all, the morphological consequences, i.e., impaired healing and increased neovascularization, in response to epithelial injury were similar whether *HO-2* expression was inhibited locally or depleted globally.

It is recognized that leukocytes are not only capable of amplifying the inflammatory response, but are also significantly involved in the repair process [44]. Gan et al. [45] showed in two rabbit models of corneal injury (Photorefractive keratectomy [PRK] and a standardized alkali corneal wound) that corneal epithelial healing rate is delayed in the absence of PMNs in vivo and PCNA expression, a marker for cell proliferation, increases in the presence of leukocytes. Other studies showed that limiting neutrophil infiltration to the cornea attenuated the inflammatory response and accelerated wound closure [46,47], whereas, exaggerated influx is linked to impaired resolution and repair and chronic inflammation as was demonstrated in the *HO-2* null mice [28,29,31]. In accordance with our previous studies, wound healing was associated with neutrophil infiltration that peaked in numbers at day 4 after injury in control shRNA-treated mice, but stayed elevated in the *HO-2* shRNA-treated mice, thus supporting the notion that *HO-2* deletion interferes with resolution.

We have previously shown that *HO-2* expression is heavily localized to the corneal epithelium, which is likely the largest source of HO activity in the uninjured cornea. During injury, however, inflammatory cells invading the cornea import HO activity; the majority of this activity appears to be driven by the inducible *HO-1* [48,49]. We have also shown that induction of *HO-1*, which constitutes a central defense response to injury, is impaired in the absence of *HO-2* [28]. The blunted *HO-1* induction in the cornea in response to injury in mice null for *HO-2* was also observed in corneas in which *HO-2* was locally suppressed. The nature of the regulatory interactions between these two isoforms, whose catalytic activity and potency are identical, is unknown. However, in view of reports showing that *HO-1* expression and activity are maximal during the resolution phase and that induction of *HO-1* expression promotes resolution whereas inhibition of HO activity is pro-inflammatory [4,27-29,33,50-52], it is reasonable to assume that this blunted response may contribute to the diminished defense against injury and consequently to the exaggerated inflammatory response in the *HO-2* shRNA-treated eyes.

Epithelial injury was also associated with a distinct increase in *MMP-2* and *MMP-9* expression. The expression of both *MMP-2* and *MMP-9* has been shown to increase in response to injury and contribute to epithelial wound repair [34,35]. On the other hand, studies with *MMP-2* null mice demonstrated decreased corneal neovascularization in response to removal of the corneal epithelium [53]. In this, and in our previous study, *MMP-2* mRNA expression appears to be intimately related to corneal neovascularization. Bellner et al. [31] showed that lingering and elevated levels of *MMP-2* mRNA in response to epithelial debridement were significantly lowered in corneas from *HO-2* null mice treated with biliverdin. In this study an increased angiogenic response was observed in the *HO-2* shRNA-treated corneas that showed a tendency of elevated levels of *MMP-2* mRNA at day 7 after injury as compared to control shRNA-treated corneas. To this end, we performed a full-scale gene microarray comparing WT and *HO-2* null corneas; preliminary data demonstrated again a close relationship between *HO-2* and *MMP-2* expression where deletion of the *HO-2* gene was associated with a sixfold increase in *MMP-2* expression (Bellner, unpublished data). The nature of this relationship with regard to corneal response to injury needs to be further explored.

It has become clear that a functioning HO system is crucial for the anti-inflammatory circuits in the cornea, and that enhancement through treatment with stannous chloride of wild type mice [27], or partial replenishment in the shape of one of its metabolic products, biliverdin [29,31]. It is possible that corneal epithelial *HO-2* plays a significant role in regulating this critical wave of neutrophils to the cornea. The local knockdown of *HO-2* impairs this ability while global knockout not only negates it but also alters the characteristics of the infiltrated cells [28]. Additional studies are needed to explore the role of corneal *HO-2* in regulating neutrophil infiltration and resolution.
